# The Effect of Herpes Simplex Virus-Type-1 (HSV-1) Oncolytic Immunotherapy on the Tumor Microenvironment

**DOI:** 10.3390/v13071200

**Published:** 2021-06-22

**Authors:** Ifeanyi Kingsley Uche, Konstantin G. Kousoulas, Paul J. F. Rider

**Affiliations:** Division of Biotechnology and Molecular Medicine, Department of Pathobiological Sciences, School of Veterinary Medicine, Louisiana State University, Baton Rouge, LA 70803, USA; Iuche1@lsu.edu

**Keywords:** herpes, VC2, immunotherapy, oncolytic virus, herpesvirus, HSV

## Abstract

The development of cancer causes disruption of anti-tumor immunity required for surveillance and elimination of tumor cells. Immunotherapeutic strategies aim for the restoration or establishment of these anti-tumor immune responses. Cancer immunotherapies include immune checkpoint inhibitors (ICIs), adoptive cellular therapy (ACT), cancer vaccines, and oncolytic virotherapy (OVT). The clinical success of some of these immunotherapeutic modalities, including herpes simplex virus type-1 derived OVT, resulted in Food and Drug Administration (FDA) approval for use in treatment of human cancers. However, a significant proportion of patients do not respond or benefit equally from these immunotherapies. The creation of an immunosuppressive tumor microenvironment (TME) represents an important barrier preventing success of many immunotherapeutic approaches. Mechanisms of immunosuppression in the TME are a major area of current research. In this review, we discuss how oncolytic HSV affects the tumor microenvironment to promote anti-tumor immune responses. Where possible we focus on oncolytic HSV strains for which clinical data is available, and discuss how these viruses alter the vasculature, extracellular matrix and immune responses in the tumor microenvironment.

## 1. Introduction

Apart from cancer cells, the tumor mass is comprised of a number of cellular and subcellular constituents. These include resident stromal cells, such as fibroblast and endothelial cells, immune cells as well as cancer-associated fibroblasts (CAFs) and extracellular matrix (ECM) proteins, collectively known as the tumor microenvironment (TME) [1]. Interactions between cancer cells and the components of the TME creates an immunosuppressive network, which profoundly influences tumor development, progression, and metastasis [2]. Importantly, immunosuppression in the TME may result in a deficient therapeutic response and resistance to treatment.

Due to the prominent role of the TME in cancer, much effort has been put into developing therapies that target components of the TME. A notable example is the clinical success of immune checkpoint inhibitors (ICIs), which function to restore anti-tumor T-cell activity [3]. Although current cancer immunotherapies including ICIs and chimeric antigen receptor T cells (CAR-T) have demonstrated promising therapeutic outcomes, limitations such as low response rate and serious side effects have been reported in some cancer patients [3,4].

Oncolytic virotherapy (OVT) represents a novel form of immunotherapy, where oncolytic viruses (OVs) are used to kill cancer cells. The lytic activity of the virus promotes release of tumor antigens and provides an immunogenic context that is thought to support the development of anti-tumor immune responses [5,6,7]. Additionally, as a combination therapy, OVT has also been shown to improve the efficacy of other immunotherapies, such as ICIs [8]. Many virus families are currently studied as oncolytic virotherapies [9]. Each has characteristics that inform their utility. Due to the extensive clinical development of herpes simplex viruses as OVT, including the only FDA approved OVT, we focus this review primarily on oncolytic HSV-1 (oHSV) OVT.

HSV-1 possess several advantages over other viruses that inform their use as OVT agents. These include: (a) its ability to infect a variety of cell types, (b) its large genome (152 kb) that allows for insertion of large or multiple transgenes, (c) it remains as an episome upon infection, precluding insertional mutagenesis, (d) it can be controlled by antiviral drugs and (e) the extensive knowledge of the molecular biology of herpesvirus infection [10].

In this review, we discuss what is known regarding how oncolytic HSV affects the tumor microenvironment to promote anti-tumor immune responses and how this knowledge may be used to inform the rational design of future, more efficacious immunotherapies.

## 2. HSV-Derived Oncolytic Viruses

The concept of using viruses for cancer treatment was conceived following observations in case reports that tumor regressions were sometimes associated with natural virus infections [11]. These anecdotal observations spawned a small number of clinical studies. A notable example was the study conducted in 1949, where 22 patients suffering from Hodgkin’s disease were treated with sera or tissue extracts containing hepatitis virus [12]. From 1950 to 1970, more studies were performed where cancer patients were administered wild type viruses, including Egypt 101 virus, adenovirus, and mumps virus [11]. Although there were often glimmers of activity in these studies, these viruses were not deemed viable cancer therapeutics. This was largely due to safety concerns and because of the limited understanding of the molecular biology of these viruses. As a result, there was a decrease in activity in the field of OVT until the emergence of recombinant DNA technology in the early 1990s [11]. This technology facilitated the development of safer OVT by allowing targeted mutations to improve the safety profiles of a number of viruses.

At the forefront of these studies, work with HSV-1 heralded the era of engineering viruses to selectively replicate in malignant cells, when Martuza et al. demonstrated first that an HSV-1 mutant, *dl*sptk, which lacks the viral gene encoding thymidine kinase (TK), efficiently killed both immortalized and short-term human malignant glioma cells in vitro [13]. Their experiments in nude mice with engrafted subcutaneous or intracranial glioma, also revealed that the intratumoral inoculation of *dl*sptk caused tumor growth inhibition and prolonged survival [13]. Importantly, the oncolytic herpes simplex virus (oHSV) *dl*sptk served as a proof of principle that attenuated mutants of HSV-1 could potentially be used for cancer therapy. However, while the deletion of thymidine kinase in the *dl*sptk virus allows it to selectively replicate in tumor cells, this virus is also not susceptible to conventional anti-herpesvirus drugs such as acyclovir [14]. Thus, other rationally designed HSV-1-derived viruses have been developed.

At present, Talimogene laherparepvec (T-VEC; Imlygic™), a double-mutated HSV-1 vector of the JS-1 strain with deletions in the infected cell protein (ICP) γ34.5 and ICP47 genes, is the only OVT approved by the United States Food and Drug Administration (FDA) and European Union (EU) for the treatment of human cancers [15,16]. TVEC™ and other oHSV will be described in more detail below. While, at present, TVEC™ is the only FDA approved OVT, there are a number of HSV-1-derived oncolytic viruses being studied in pre-clinical and clinical settings. These viruses use different attenuation and delivery strategies as well as possess a number of transgene insertions many of which are expected to alter their interactions with the TME.

## 3. Tumor Microenvironment

**Figure 1:**
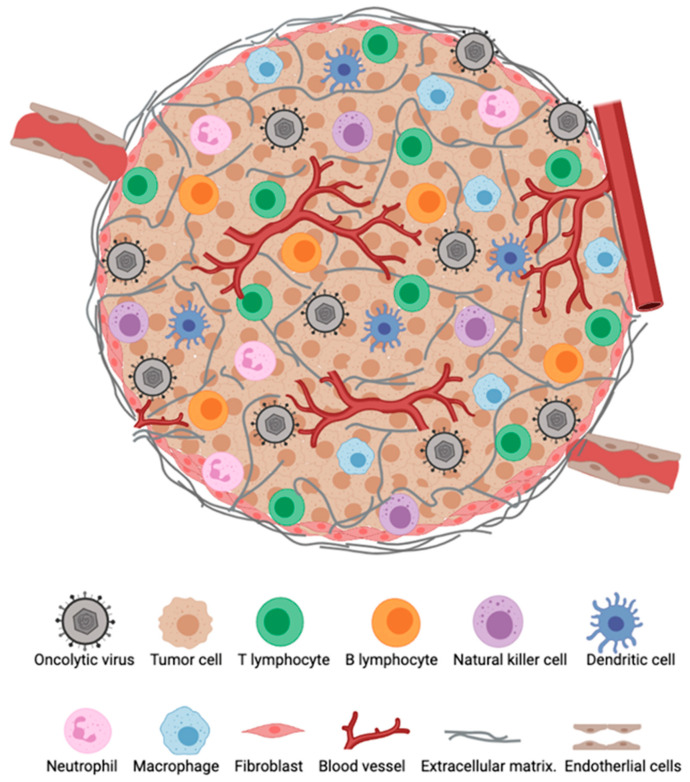
The tumor microenvironment (TME). Major cellular constituents of the TME during oncolytic virotherapy (OVT).

The TME represents an important barrier that can hinder immune responses against tumors and also attenuate immunotherapeutic efficacy (Figure 1). In situ immune components of tumors, including T cells, B cells, natural killer cells (NK), dendritic cells (DCs), macrophages, granulocytes as well as suppressive regulatory cells subtypes such as regulatory T cells (Tregs), regulatory B cells (Bregs), and myeloid-derived suppressor cells (MDSCs), play crucial roles in the host cancer immunosurveillance system, although their contribution varies between tumor types and among patients with the same cancer [17]. The type, density, and location of immune infiltrates within a tumor have been suggested to have a clinical prognostic impact [18]. Importantly, the infiltration of T cells, particularly CD8+ T cells, have has been shown to correlate with augmented responses to immunotherapy and improved survival [19,20,21].

Tumors can be classified as immunologically “hot” or “cold” [20,22]. “Hot” or inflamed tumors have an abundance of infiltrating T cells and molecular signatures of immune activation and are usually responsive to immunotherapy [17]. By contrast, “cold” tumors are defined to have limited number of infiltrating T cells and are not sufficiently primed for immune recognition [17]. These cold tumors often contain Tregs, Bregs, and MDSCs, which prevent cytotoxic immune cells from penetrating into the TME [17]. Therefore, turning a cold tumor hot is essential and may significantly enhance the therapeutic efficacy of cancer immunotherapies. Unfortunately, only a minority of patients with hot cancers seem to respond and benefit from immunotherapies like checkpoint blockers, cancer vaccines and CAR-T cells. To improve clinical outcomes in patients with a variety of cancer types, significant efforts are currently undertaken to combine immunotherapies themselves or with traditional cancer therapies, such as chemotherapy, radiation therapy, or surgery [20].

The generation of an anti-tumor response involves a cyclic process referred to as the cancer-immunity cycle [23]. This cycle begins with liberation of neoantigens generated as a result of spontaneous immunogenic cell death, chemotherapy, radiation, and/or OVT among others. The released neoantigens are captured and processed by antigen-presenting cells (APCs), which migrate to the draining lymph nodes to prime and activate tumor-specific T cells. Tumor-specific T cells traffic to and infiltrate the tumor bed where they recognize and kill cancer cells. The killing of cancer cells releases additional tumor antigens, fueling the cancer immunity cycle.

## 4. Tumor Microenvironment and Oncolytic Virotherapy

### 4.1. oHSV and Anti-Tumor Immune Responses

A number of candidate oHSVs are currently in development [24]. Our group and others have demonstrated the significant impact of oHSV OVT on the development of anti-tumor immune responses in pre-clinical or clinical studies [25,26,27,28]. Specifically, these studies have highlighted the ability of oHSVs to change the TME from “cold” or immunosuppressive to “hot” or immunostimulatory as a component of their efficacy. Given the central role of the TME in controlling the development of anti-tumor immune responses, understanding the cellular and molecular changes in the TME during OVT would inform the development of more efficacious immunotherapies for the treatment of human and animal cancers. While the pre-clinical pipeline of oHSV is rich with promising candidates [25,26,27,28,29], we focus in this section on several oHSVs for which there is clinical data.

T-VEC™ is the first FDA approved OVT for treatment of patients with unresectable melanoma [15]. T-VEC is a modified HSV-1 OV derived from the JS-1 strain in which two copies of its ICP34.5 genes which encodes the neurovirulence factor are deleted [16]. The ICP34.5 gene is also important for viral replication, viral egress from cells and prevention of cellular block to viral protein synthesis in infected cells through its inhibitory activity on the protein kinase R (PKR) pathway [30,31,32]. PKR stops protein synthesis by phosphorylating the eukaryotic translation initiation factor 2 alpha (eIF2α) [32]. The ICP34.5 protein redirects protein phosphatase 1α (PP1α), which reverses the phosphorylation of eIF2α to restore protein synthesis [32]. Deletion of the ICP34.5 gene decreases pathogenesis and prevents replication of the virus in normal cells. In addition, T-VEC contains a deletion in the gene encoding the ICP47 protein [16]. HSV-1 uses the ICP47 protein to inhibit transporter associated with antigen presentation (TAP), thus it downregulates antigen presentation through MHC Class I, as a way of evading immune response during infection [33]. The deletion of the ICP47 gene increases antigen presentation and enhances viral immunogenicity. Additionally, ICP47 inactivation in T-VEC puts the herpes unique short 11 (US11) under immediate early promoter control which results in enhanced and earlier expression of US11, whose gene product inhibits PKR activity [34]. Finally, two copies of the human granulocyte–macrophage colony stimulating factor (GM-CSF) gene are inserted in place of the deleted ICP34.5 gene loci [16]. GM-CSF functions as a potent immune stimulator by promoting the recruitment and maturation of DCs and macrophages [16]. It is not clear what contribution the GM-CSF makes to anti-tumor efficacy of T-VEC™ as other vectors have shown efficacy in the absence of cytokine transgenes [25].

T-VEC demonstrated strong lytic activity when tested in vitro in human tumor cell lines, including melanoma and pancreatic cancer cells [16]. In mice, intratumoral injection of A20 lymphoma tumors with T-VEC resulted in significant reduction of tumor volume of both injected and non-injected tumors, and treated mice rejected subsequent tumor re-challenge [16]. Corroborating the aforementioned study, a recent study in mice found similar systemic immune responses induced by T-VEC to distal untreated tumors [35]. Furthermore, a significant increase of CD3+ and CD8+ T cells was observed in both injected and contralateral tumors and these T cells were tumor specific [35]. In humans, TVEC monotherapy has also demonstrated distant responses in untreated tumors [36]. Intralesional injection of T-VEC altered the TME and increased MART-1 (melanoma antigen recognized by T-cells 1) specific CD8+ T-cell infiltration in melanoma patients [36]. There was also evidence of these cytotoxic T cells in the peripheral blood of treated patients, suggesting the induction of systemic anti-tumor immunity [8,36]. In addition, injected lesions demonstrated a decrease in Tregs, T-suppressor cells, and MDSCs [36]. In another study, T-VEC in combination with anti-PD-1 antibody, pembrolizumab, altered the TME and increased cytotoxic CD8+ T cell infiltration [8]. These findings demonstrate that OVT can improve the efficacy of ICIs as a combination therapy.

The G207 strain is an attenuated HSV-1 OV based on the HSV-1 (F) strain, which contains deletions of both copies of the ICP34.5 gene, as well as an *Escherichia coli* lacZ gene insertion which inactivates the ICP6 gene [37]. The ICP6 gene encodes for a subunit of ribonucleotide reductase, an enzyme necessary for viral DNA synthesis in quiescent cells [38]. The inactivation of the ICP6 gene restricts the virus to replicate in actively dividing cells, thus giving the virus selectivity for tumor cells [37].

Early reports demonstrated the utility of G207 as an in situ cancer vaccine [39]. In this study, G207, after intratumoral inoculation of colorectal carcinoma cells, was reported to induce an anti-tumor immune response. Further, in mice that had two tumors engrafted, intratumoral inoculation with G207 reduced tumor growth rates in treated and contralaterally engrafted but untreated tumors, demonstrating an abscopal effect [39]. Intratumoral inoculation with G207 in pre-clinical models of U-87MG gliomas resulted in a significant reduction of tumor volume and prolonged survival [37]. Tumor vasculatures were destroyed in mice bearing rhabdomyosarcoma xenografts following intratumoral treatment with G207 [40]. In the same line, work by Huszthy et al. in athymic nude rats implanted with human glioblastoma (GBM) biopsies further suggests that G207 may possess antiangiogenic properties [41]. The authors also observed accumulated CD68+ microglia cells and macrophages within the tumor bed around viral plaques in G207 injected tumors [41]. In this study, viral plaques and CD68+ cells within the tumor tissue were confirmed by immunostaining [41].

In Phase 1b clinical trials with patients suffering from recurrent GBM, G207 was introduced directly into resected cavities after tumors were removed surgically [42]. G207 demonstrated high safety profiles with post treatment evidence of significant infiltration of CD8+ T cells and macrophages/microglia into the TME [42]. Most recently, results from the first in-human trial of G207 for malignant pediatric cerebellar brain tumors were reported [43,44]. Patients had catheters introduced which allowed direct administration of virus to tumors. Results were highly encouraging with only a single non-responder of 12 patients enrolled. There was substantial evidence of lymphocyte infiltration into tumors in some patients [44]. Interestingly, when patients who were HSV-1 seropositive were excluded from analysis, increased circulating numbers of natural killer (NK) cells was found to positively correlate with survival. Median survival for front-line therapies of high-grade pediatric gliomas is 5.6 months [45,46]. While not directly comparable, in this trial, G207 treatment resulted in median survival of 12.2 months [23]. Moreover, at the time of reporting four of the patients were still surviving 18 months after treatment with G207. This result exceeded expectations and provides strong support for Phase 2 trials.

HSV1716 is derived from HSV-1 strain 17 and lacks both copies of the ICP34.5 genes [47]. HSV1716 OVT has been demonstrated to reduce tumor growth and provide survival advantage as well as to induce significant tumor infiltration of inflammatory immune cells, including CD4+ T cells, CD8+ T cells, NK cells and macrophages in mouse models of intracranial M-3 S91 Cloudman melanoma, 4T1 breast, and rhabdomyosarcoma cancers [48,49,50]. One mechanism used by HSV1716 to recruit effector immune cells into the TME is through the upregulation of chemokines: IFN-γ-inducible protein-10 (IP-10 also known as CXCL10) and monokine induced by IFN-γ (MIG also known as CXCL9) [51]. IP-10 and MIG are known to have chemotactic effects on activated T cells and NK cells. Recent studies in PyMT-TS1 breast cancer model further demonstrated HSV1716’s ability to alter the TME immunosuppressive milieu. In this study, HSV1716 OVT resulted to reduced number of Tregs and reprogrammed M2-like TAMs to a more pro-inflammatory M1-like phenotype in the TME when compared to matched controls [52].

HSV1716 has been evaluated in a number of clinical trials as an intervention strategy for melanoma, mesothelioma, non-CNS solid tumors, and glioma [53,54,55,56]. For mesothelioma, patients were administered HSV1716 via an intrapleural catheter [56]. In the majority of patients after treatment with HSV1716, levels of Th1 cytokines IL-2, IFN-g, TNFa in pleural fluid were increased 5–10-fold [56]. Interestingly, the authors reported the development of novel anti-tumoral antibody responses in four of the patients treated with HSV1716 [56]. In a previous study, HSV1716 was administered intravenously in young patients with extra-cranial solid tumors [54]. Impressively, intravenous administration at up to 10^7^ infectious doses was well tolerated and two patients exhibited stable disease. Unfortunately, TMEs were not studied in biopsied specimens. In another study in young patients with brain tumors, up to 10^7^ infectious units were administered intratumorally [54]. Administration was well tolerated. No biopsies were performed, but imaging revealed metabolic activity consistent with inflammatory responses.

HF10 is a non-engineered, spontaneously mutated OV derived from the HSV-1 HF strain. At the genome level, HF10 lacks the expression of functional unique long (UL) 43, UL49.5, UL55, UL56, and latency-associated transcripts (LAT), and overexpresses UL53 and UL54 genes [57]. The functions of these altered genes and how they may contribute to the antitumor efficacy of HF10 have been reviewed [57]. In a C3H mouse model of head and neck squamous cell carcinoma (SCC VII), intratumoral injection of HF10 reduced tumor growth and significantly enhanced survival [58]. Immunohistochemistry analysis showed evidence of tumor necrosis and infiltration of CD8+ T cells around HSV-infected cells in HF10 treated tumors [58]. These findings correspond with the results of previous studies of HF10 OVT against other malignancies [59], however, the authors did not show whether HF10 as a monotherapy, altered the immunosuppressive cell populations in the TME.

There have been several clinical trials to evaluate the safety and efficacy of HF10 for a number of different cancers including head and neck squamous cell carcinoma (HNSCC), breast cancer, melanoma and pancreatic cancer [57,60,61,62,63,64]. All trials report that HF10 was safe and tolerable in each trial. Analysis of infiltrating immune cells was reported in a subset of these trials. In a recent Phase I trial of nonresectable pancreatic cancer where patients were treated intratumorally with HF10, immunohistochemistry on HF10 treated tumors revealed a statistically significant infiltration of CD8+ T-cells compared to tumors that did not receive HF10 [64]. Analysis of CD4+ T-cells revealed no statistically significant differences between treated and untreated tumors. The study also included analysis of macrophages and other APCs. Of these, macrophages were found to infiltrate tumors in statistically significant numbers in HF10 treated tumors relative to untreated tumors. Consistent with a trial for G207 [44], the authors noted an increase in circulating NK cells in patients treated with HF10.

NV1020 is derived from the HSV-1 strain F designated as R7020, that was originally developed as a vaccine against HSV-1 and HSV-2 infection [65]. The NV1020 genome contains a 15-kb deletion region at the UL/S junction, which encompasses genes encoding ICP0, ICP4, and ICP34.5, as well as UL56 [66]. NV1020 is further attenuated by a 700-bp deletion in the *tk* gene locus that prevents the expression of the overlapping UL24 gene [66]. In addition, the virus carries an insertion of an exogenous copy of the HSV-1 *tk* gene, and a 5.2-kb fragment of HSV-2 DNA [66]. NV1020 replicates efficiently in transformed cells and the insertion of the *tk* gene ensures that viral infection or toxicity can be controlled with antiviral drugs, like acyclovir.

The safety and efficacy profile of NV1020 in the treatment of peritoneally disseminated gastric cancer has previously been investigated in preclinical models. When tumor bearing mice were treated intraperitoneally with NV1020, it significantly reduced tumor burdens and conferred significant survival advantage when compared to control treated animals [67]. In addition, when the brain, liver, kidneys and tumor of NV1020 treated mice were evaluated for HSV biodistribution and necrosis, immunohistochemistry analysis showed no virus or necrosis in any non-tumor tissues [67]. However, significant HSV staining and extensive necrosis was observed in tumor specimens. Furthermore, NV1020 has also been shown to have significant promise in the treatment of other pre-clinical cancer models, including pancreatic, pleural, and bladder cancer, as well as head and neck squamous cell carcinoma [68,69,70,71].

The safety and tolerability of NV1020 was first evaluated in a Phase 1 study involving patients with colorectal cancer metastatic to liver (mCRC) [72]. NV1020 was administered to 12 patients (3 per cohort) via intrahepatic arterial infusion in escalating dose of 3 × 10^6^, 1 × 10^7^, 3 × 10^7^, and 1 × 10^8^ PFU. All patients received cycles of floxuridine through a chemotherapy infusion pump with several combinations of chemotherapy (irinotecan, 5-fluorouracil, leucovorin, or oxaliplatin) 1 and 2 months after administration of virus. Most frequent adverse events associated with the administration of NV1020 included pyrexia, headache and rigor. Although, one patient had a severe case of increased gamma glutamyl transferase (GGT) levels occurring 12 h after virus infusion. No significant change in serum levels of cytokines and T cells due NV1020 administration was observed. Radiologic assessment of anti-tumor activity 28 days after viral administration showed that two patients had reduction in tumor size of 39% (1 × 10^8^ PFU cohort) and 20% (3 × 10^7^ PFU cohort), respectively. In addition, 3 patients demonstrated progression of disease, while 7 patients had disease stabilization.

A subsequent study aimed to evaluate the therapeutic effects of an optimally tolerated dose of NV1020 in patients with mCRC has been completed. According to the results, treatment with virus through hepatic artery infusion was well tolerated and was associated with stable disease in 50% of the patients. Immunologically, individual infusions of NV1020 induced a dose-related increase in the levels of IL-6, TNF-α, and IFN-γ. The addition of chemotherapy resulted to a median time to progression (TTP) and a median overall survival (OS) of 6.4 months and 11.8 months respectively. Altogether, this study suggests that NV1020 can stabilize liver metastases and sensitize tumors to chemotherapy resulting prolonged overall survival in patients with mCRC [73].

### 4.2. Interactions of oHSV with the Tumor Vasculature

Tumor vasculatures restrain the migration of immune cells from reaching tumor targets through the expression of immunosuppressive ligands and the downregulation of adhesion molecules [74]. Studies on how oHSV-derived OVT disrupts tumor-induced vasculature has demonstrated mixed results. On one hand, studies have shown a direct anti-angiogenic effects of oHSV [75]. Although most of the reported anti-angiogenic oHSV vectors are armed with angiostatic factors or angiogenesis inhibitors. One example is the oHSV RAMBO (rapid antiangiogenesis mediated by oncolytic virus). Hardcastle et al. hypothesized that incorporating an extracellular fragment of the brain-specific angiogenesis inhibitor 1 (BAI1), vasculostatin (Vstat120), in RAMBO would counter the downregulation of thrombospondin 1 (TSP-1) and the increased cysteine-rich 61 (CYR61) integrin activation in the TME, thereby enhancing the anti-tumor efficacy [76]. Indeed, RAMBO treatment promoted significant anti-tumor and anti-angiogenic responses over the control virus, in the treatment of mice bearing intracranial and subcutaneous gliomas. In addition, RAMBO-treated tumors showed a significant reduction in tumor microvessel density (MVD) and vascular volume fraction (VVF) [76]. Similarly, Tsuji et al. demonstrated that treatment with T-TSP-1, an oHSV armed with human TSP-1, reduced tumor MVD and exerted an enhanced anti-tumor efficacy against human gastric cancer in vivo [77].

On the other hand, other reports have suggested that oHSVs induce pro-angiogenic responses [78,79]. The downregulation of TSP-1, an anti-angiogenic factor, and the increased expression of CYR61, a pro-angiogenic factor, in infected cells are some of the limitations of the neovascular responses associated with oHSV infection [78,79]. When taken together, there is no clear census regarding the anti-angiogenic or pro-angiogenic nature of oHSV vectors.

### 4.3. Interactions of oHSV with the Extracellular Matrix of Tumors

The extracellular matrix (ECM) of tumors controls a number of important regulatory processes central to tumor pathology [80]. However, its function as a barrier to prevent viral replication and spread has motivated a number of groups to manipulate the ECM as a component of OVT [81,82,83,84,85,86]. These efforts include the exogenous addition of enzymes to degrade the ECM during OVT and the engineering of novel viruses that express matrix degrading enzymes. The molecular makeup of the ECM can be unique to each tumor. As such, the approaches to disrupt the ECM as a component of OVT may not represent a “one size fits all” solution. Further, ECM remodeling proteins such as matrix metalloproteinases (MMPs), while generally understood to be protumoral, can have anti-tumoral effects depending on individual tumor characteristics [87]. Bearing this in mind, a number of groups have been successful incorporating ECM-targeting approaches for OVT.

Building on earlier work that demonstrated exogenous addition of matrix metalloproteinase 9 (MMP9) in neuroblastoma cells enhanced oHSV spread [81], Sette et al. created a novel oHSV that expresses MMP9 (KMMP9) [88]. MMP9 degrades Type IV collagen which is present in the ECM of glioblastoma multiforme (GBM). KMMP9 possessed enhanced spread in GBM spheroids and treatment of intracranial mouse xenografts with KMMP9 resulted in increased survival compared to xenografts treated with control non-MMP9 expressing-virus [88].

Mckee et al. demonstrated that targeting collagen using bacterial collagenases enhanced efficacy of OVT in melanoma xenograft models [84]. Bacterial collagenases were introduced intratumorally, coincident with MGH2, an oHSV vector. The combined effect was enhanced intratumoral spread of virus and significantly reduced tumor growth rates.

In brain tumors many CSPGs are upregulated and this upregulation is associated with the enhanced pathogenicity of these tumors. Sugar modifications on CSPGs are responsible for limiting diffusion in the ECM. Dmitrievea et al. reasoned that removal of these moieties with a bacterial enzyme, chondroitinase ABC (chase-ABC), would facilitate replication and spread of oHSV through CNS tumors [83]. To this end, they engineered an oHSV (OV-Chase) to express Chase-ABC. OV-Chase significantly enhanced replication and spread through subcutaneous gliomas, reduced tumor growth rates and increased survival in mouse glioma xenografts.

More direct approaches have been taken to subvert the ECM barrier to viral replication and spread through tumors. E-cadherin is a molecule that facilitates cellular adhesion through nectin-1. Nectin-1 is the predominant receptor for HSV-1 infection and the addition of E-cadherin was hypothesized to directly increase viral infection and spread by promoting receptor binding [82]. This group engineered an oHSV (OVCDH1) to express human E-cadherin. OVCDH1 exhibited enhanced spread through GBM cells in vitro, reduced tumor growth rates in vivo and enhanced survival in xenograft and immunocompetent models of GBM.

Most studies of the effect of manipulating the ECM on OVT efficacy have focused on viral replication and spread. There has been relatively little effort spent to look at the effect manipulating the ECM during OVT has on immune cell infiltration. The above study is particularly interesting in this respect, as they examined the effect of E-cadherin on infiltrating NK cells, macrophage, microglia and T-cells. Perhaps due to an increase in cytolytic activity of OVCDH1 this group noted an increase in NK cell infiltration into tumors after OVCDH1 treatment versus control virus treatment. No other differences in immune cell infiltration were observed between OVCDH1 and control groups.

Finally, while the above groups demonstrated that overexpression of ECM proteins can benefit OVT efficacy, Mahller et al., reasoned that inhibition of ECM protein activity may benefit OVT [85]. This group hypothesized that, as the function of many ECM protein activities are pro-tumoral, using a virus to express TIMP-3, a matrix metalloprotein inhibitor, might benefit oHSV-derived OVT. TIMP-3, tissue inhibitor of MMPs 3, is an inhibitor of all MMPs. Treatment with TIMP-3 reduced tumor growth rates in a number of cancer models [89,90,91]. Using this rationale, Mahller et al., engineered the G207 oHSV (described below and in Table 1) to express TIMP-3 and called it rQT3. Compared to control virus which expresses luciferase, treatment of neuroblastoma and malignant peripheral nerve sheath (MPNST) xenografts with rQT3 was superior at reducing tumor growth rates and enhancing survival.

## 5. oHSV and Immune Checkpoint Inhibitors (ICI)

A major mechanism through which the TME creates a hostile environment for immune cells is by inducing the upregulation of surface inhibitory receptors such as cytotoxic T-lymphocyte-associate protein 4 (CTLA-4), and programmed death-1 (PD-1), as a mechanism for tumors to avoid immune surveillance. CTLA-4 blocks T cell activation, by counteracting CD28-mediated costimulatory signaling, by binding to B7 ligands expressed on APCs [96]. CTLA-4 is also constitutively expressed on Tregs, which contributes to the immunosuppression of the TME [3]. PD-1 is expressed on activated T cells and binds to one of two ligands, program death-ligand 1 (PD-L1) and PD-L2. Numerous tumors express PD-L1, which when bound to PD-1, attenuates T cell functions such as proliferation, cytokine production, and cytotoxicity [97,98]. Lymphocyte activation gene-3 (LAG-3), T cell immunoglobulin domain and mucin domain-3 (TIM-3), and T cell immunoglobulin and ITIM domain (TIGIT) are other immune checkpoints known to inhibit effector T cell responses [99].

The blockade of the PD-1/PD-L1 and/or CTLA-4/B7 inhibitory pathways by ICIs can reinvigorate the effector functions of T cells in the TME. However, limited response rates and treatment related toxicities has been reported with ICIs [3,100]. Heterogenicity between cancer types, and the lack of sufficient immune activation are some of the reasons for low response rates. Thus, it is reasonable that the outcome of ICI therapeutic intervention can be improved as a combinatory therapy with other treatment modalities such as OVT. In this sense, OVs not only lyse the tumors cells but also alters the TME into a highly immunogenic one, which has been suggested to favor the efficacy of immune checkpoint blockades. For example, in a syngeneic murine rhabdomyosarcoma model, combining oHSV HSV1716 with PD-1 blockade significantly prolonged survival compared to PD-1 blockade or HSV1716 treatments alone [50]. In addition, the therapeutic outcomes of the combinatory therapy correlated with increased tumor infiltration of CD4+ and CD8+ T cells, but not Tregs, compared to the control groups [50]. Similarly, when Saha et al. tested a triple combination of anti-PD1, and anti-CTLA4 antibodies with an oHSV expressing murine IL-12 (G47∆-mIL-12) in mouse GBM models, they observed that the treatment produced durable cures in most mice [101]. The dual combination of anti-CTLA4, and anti-PD1 increased median survival by 37% compared to 20% observed for anti-CTLA-4 alone. However, the addition of G47Δ-mIL12, with anti-CTLA4, and anti-PD1 led to 89% long-term survivors. Analysis of tumor-associated immune cell infiltrates showed that the treatment led to reduction of Tregs, influx of M1-like macrophages, increased infiltration of CD8+ T cells, and increased T effector to T regulatory cell ratios. Furthermore, when macrophage, CD4, or CD8+ T cells were depleted, the efficacy of the treatment was impaired, suggesting that are they are required for efficacy [101].

Concordant with these findings in murine studies, the importance of combining ICIs with oHSV-derived OVT in clinical settings has also been demonstrated. In a Phase 1b clinical trial with patients with advanced melanoma, anti-PD-1antibody (pembrolizumab) plus T-VEC combination led to an increased tumor infiltration of IFN-γ+ CD8+ T cells in patients with tumors with low level of immune cell infiltrates and negative IFN-γ signatures [8]. This combination therapy resulted in a high objective response rate (ORR) of 62% (with a 33% complete response) compared with prior single agent pembrolizumab therapy. Data from another clinical study which tested the combination of CTLA-4 blockade (ipilimumab) with T-VEC in melanoma patients, showed that the combination was well tolerated and had greater anti-tumor efficacy than ipilimumab monotherapy [102]. According to the results, the ORR was significantly higher for the combination therapy than for ipilimumab alone (39% vs. 18%, respectively, *p* = 0.002). Taken together, these promising pre-clinical and clinical results suggest that OVT can sensitize immunologically cold tumors to ICIs.

## 6. Conclusions and Future Directions

Studies have shown that hot or inflamed tumors, which contain an abundance of infiltrating T cells in the TME, are more likely to respond to immunotherapies, as compared with cold immune desert tumors, which have low or no T cells [17]. Thus, strategies to turn cold tumors hot and hence promote anti-tumor immunity hold great promise. One such strategy includes OVT, a novel form of cancer immunotherapy.

Although with the FDA approval of T-VEC together with the promising results yielded by other oHSVs in clinical trials, there are still many obstacles that needs to be tackled in the development of additional improved HSV-1 OVTs. The first challenge is choosing an optimal OVT, as a single OV may not be effective in all types of cancers. Many HSV-1 OVs are engineered to replicate in actively dividing cells because it has been proposed that cancer cells are actively replicating while healthy cells are not. Unfortunately, not all cancer cells within a tumor mass undergo active replication [103]. In addition, the TME contains non-tumoral cells such as immune infiltrates, fibroblast, and endothelial cells, which have been shown to not support the replication of OVs designed according to the aforementioned principles [104,105]. More recent approaches to restrict HSV-1 OVs to cancer cells include receptor retargeting [106], use of specific promoters to express critical viral genes [107], transcriptional and translational regulation of essential viral gene expression [108], and incorporation of miRNA target sites into viral genes [109]. However, all of these approaches possess limitations, including the possibility of reversion of attenuation, increased toxicity, in addition to over-attenuation of viral replication and thus efficacy. Hence, an attractive approach for the development of next generation HSV-1 OVTs might focus on maintaining much of viral replication potential, while eliminating their ability to spread to the nervous system, where they can establish latency, possibly recombine with wild-type virus strains, or reactivate and subsequently cause disease. Our laboratory has recently identified mutations that blocks HSV-1 entry into neurons [110]. This novel oHSV, VC2, replicates to similar viral titers to its parental HSV-1 F strain virus, but is not neuroinvasive, and in a B16F10-derived mouse melanoma model, demonstrated significant efficacy and altered the immunosuppressive TME [25].

Optimizing efficient HSV-1 OV delivery systems is another challenge that requires further studies. Intratumoral delivery is the most common route of oHSV delivery in many pre-clinical and clinical studies. This method directly targets the OV into the injected tumor and bypasses viral dilution in blood, antiviral immunity or sequestration in non-targeted cells or tissues. Intratumoral delivery is suitable for cutaneous or subcutaneous lesions treatment, although safety issues can arise for deep visceral lesions or those located in the brain, especially when repeated dosing is needed [111]. Utilization of image-guidance techniques may be a promising approach to maximize oHSV delivery to remote inaccessible lesions [112]. On the other hand, intravenous delivery represents an ideal route of OV administration as it enables broad virus distribution to both primary and metastatic tumors regardless of location. In addition, this route is relatively noninvasive, and inoculations can be frequently repeated [103]. However, for HSV-1 OVs, pre-existing immunity is a concern as the virus may be rapidly cleared by pre-existing circulating neutralizing antibodies before it reaches its target tissues. Studies from our laboratory and other groups have demonstrated that prior immunity to HSV-1 does not have an effect on the efficacy of HSV-1 OVT in mice [16,25] or humans [15], although others have reported that it did enhanced therapeutic outcome in vivo [48]. It has been suggested that the latter may be true, particularly for herpesviruses, which as part of their replication strategy periodically reactivate and spread, causing continued activation of robust adaptive immune responses [113]. Therefore, rather than suppressing HSV-1 derived OVTs, pre-existing immunity may enhance their efficacy [113]. However, more studies are needed as there are limited efficacy data from intravenous oHSV trials to judge. Nevertheless, a novel approach that can be used to bypass pre-existing immunity and secure the systemic delivery of HSV-1 OVs may involve the use of carrier cells that have tropism for tumor-bearing tissues and in addition can protect the virus from neutralization [114].

While the clinical studies performed so far are encouraging at this point it is difficult to relate the clinical outcomes to any of the observations of activity in the TME. While we and others have shown evidence of the induction of anti-tumor immune responses in animal models it is less clear in human patients, whether the lymphocyte infiltrates after oHSV OVT are anti-viral or anti-tumoral. However, it is clear that oHSV OVT is capable of significant alteration of infiltrating immune cells, as well as circulating immune cells that would be expected to exert positive anti-tumor immune responses. Beyond the scope of this review is a number of oHSV trials that are being carried out in combination with established cancer therapeutics and immunotherapeutics. Finally, it will be important to try and identify the immunological correlates to efficacy as these will inform not only the rationale design of novel oHSV but both patient selection as well as selection of therapeutics to pair with oHSV OVT.

## Figures and Tables

**Table 1:** Summary of HSV-derived Oncolytic Viruses Discussed.

Oncolytic Herpes Simplex Virus	Strain	HSV Gene Mutations	Transgene	Reference
KMMP9	KOS	Deletion of residues 2–24 and amino acid substitution, Y38C, in gD. gB:NT	Human EGFR, miR-124 target in ICP4 3′UTR, MMP9	[88]
rQT3	F	ICP34.5Δ, ICP6Δ	Human TIMP3	[92]
MGH2	F	ICP34.5Δ, ICP6Δ	CYP2B1 and human shiCE	[93,94]
OVCDH1	Q1	ICP34.5Δ, ICP6Δ	Human CDH1	[82]
OV-Chase	F	ICP34.5Δ, ICP6Δ	Bacterial Choindroitinase ABC	[83]
T-VEC	JS-1	ICP34.5Δ, ICP47Δ	Human GM-CSF	[16]
G207	F	ICP34.5Δ	*Escherichia coli* lacZ gene at ICP6	[37]
HSV1716	17	ICP34.5Δ	None	[47]
HF10	HF	Reduced expression of UL43, UL49.5, UL55, UL56 and LAT genes; increased expression of UL53, and UL54 genes	None	[57]
NV1020	F	Joint deletion (1 copy of ICP0, ICP4, ICP 34.5), UL56Δ, UL5/6 duplication, UL24Δ, TKΔ	TK under ICP4 promoter control, 5.2-kb fragment of HSV-2 DNA	[66]
G47Δ-mIL-12	F	ICP34.5Δ, ICP6Δ, ICP47Δ	Mouse IL-12	[95]
VC2	F	gKΔ, UL20Δ	None	[25]
RAMBO	F	ICP34.5Δ, ICP6Δ	Human Vstat120	[76]
T-TSP1	F	ICP34.5Δ, ICP6Δ, ICP47Δ	Human TSP-1	[77]
